# Prevalence, Type, and Molecular Spectrum of *NF1* Mutations in Patients with Neurofibromatosis Type 1 and Congenital Heart Disease

**DOI:** 10.3390/genes10090675

**Published:** 2019-09-04

**Authors:** Valentina Pinna, Paola Daniele, Giulio Calcagni, Lucio Mariniello, Roberta Criscione, Chiara Giardina, Francesca Romana Lepri, Hossein Hozhabri, Angela Alberico, Stefania Cavone, Annunziata Tina Morella, Roberta Mandile, Francesca Annunziata, Niccolò Di Giosaffatte, Maria Cecilia D’Asdia, Paolo Versacci, Rossella Capolino, Pietro Strisciuglio, Sandra Giustini, Daniela Melis, Maria Cristina Digilio, Marco Tartaglia, Bruno Marino, Alessandro De Luca

**Affiliations:** 1 UOS Diagnosi Genetica Molecolare, Fondazione IRCCS Casa Sollievo della Sofferenza, 71013 San Giovanni Rotondo (FG), Italy (V.P.) (P.D.) (R.C.) (C.G.) (H.H.) (A.A.) (S.C.) (A.T.M.) (F.A.) (N.D.G.) (M.C.D.); 2 Department of Pediatric Cardiology and Cardiac Surgery, Bambino Gesù Pediatric Hospital and Research Institute, 00165 Rome, Italy; 3 Department of Translational Medical Science, Section of Pediatrics, Federico II University, 80100 Naples, Italy (L.M.) (R.M.) (P.S.) (D.M.); 4 Department of Pediatrics, Sapienza University of Rome, 00161 Rome, Italy (P.V.) (B.M.); 5 Genetics and Rare Diseases Research Division, Ospedale Pediatrico Bambino Gesù, IRCCS, 00146 Rome, Italy (F.R.L.) (R.C.) (M.C.D.) (M.T.); 6 Department of Dermatology and Venereology, Sapienza University of Rome, Policlinico Umberto I, 00161 Rome, Italy

**Keywords:** neurofibromatosis type 1, congenital heart disease, pulmonary valve stenosis, non-truncating mutation, Noonan syndrome

## Abstract

The aim of this study was to assess the prevalence and type of congenital heart disease (CHD) and the associated mutation spectrum in a large series of patients with neurofibromatosis type 1 (NF1), and correlate the mutation type with the presence and subgroups of cardiac defects. The study cohort included 493 individuals with molecularly confirmed diagnosis of NF1 for whom cardiac evaluation data were available. CHD was reported in 62/493 (12.6%) patients. Among these patients, 23/62 (37.1%) had pulmonary valve stenosis/dysplasia, 20/62 (32.3%) had mitral valve anomalies, and 10/62 (16.1%) had septal defects. Other defects occurred as rare events. In this NF1 subcohort, three subjects carried a whole-gene deletion, while 59 were heterozygous for an intragenic mutation. A significantly increased prevalence of non-truncating intragenic mutations was either observed in individuals with CHD (22/59, 37.3%) or with pulmonary valve stenosis (13/20, 65.0%), when compared to individuals without CHD (89/420, 21.2%) (*p* = 0.038) or pulmonary valve stenosis (98/459, 21.4%) (*p* = 0.002). Similarly, patients with non-truncating *NF1* mutations displayed two- and six-fold higher risk of developing CHD (odds ratio = 1.9713, 95% confidence interval (CI): 1.1162–3.4814, *p* = 0.0193) and pulmonary valve stenosis (odds ratio = 6.8411, 95% CI: 2.6574–17.6114, *p* = 0.0001), respectively. Noteworthy, all but one patient (19/20, 95.0%) with pulmonary valve stenosis, and 18/35 (51.4%) patients with other CHDs displayed Noonan syndrome (NS)-like features. Present data confirm the significant frequency of CHD in patients with NF1, and provide further evidence for a higher than expected prevalence of *NF1* in-frame variants and NS-like characteristics in NF1 patients with CHD, particularly with pulmonary valve stenosis.

## 1. Introduction

Neurofibromatosis type 1 (NF1, MIM #162200), also known as von Recklinghausen disease, is one of the most common autosomal dominant disorders with multisystem involvement. NF1 affects approximately 1/2000 live births [1], and is characterized by considerable inter- and intra-familial clinical variability [2]. Major features of the disease include café-au-lait spots (CaLS), skinfold freckling (SF), Lisch nodules (LN), neurofibromas (NF), typical bone abnormalities, and optic pathway glioma (OPG) [2]. Diagnosis is based on the criteria defined at the National Institutes of Health (NIH) 1988 NF consensus conference [3].

NF1 is caused by heterozygous mutations of the *NF1* gene, which is located on chromosome 17q11.2, and contains 57 constitutive and three alternatively spliced exons [4,5]. *NF1* encodes neurofibromin, a protein with tumor suppressor function belonging to the family of GTPase activating proteins (GAPs). Neurofibromin is expressed in several cells, including neurons, and Schwann and glial cells, and has an important modulatory role in the control of cellular proliferation, differentiation, apoptosis, and migration. Indeed, promoting the conversion from active GTP-bound RAS to inactive GDP-bound RAS, neurofibromin negatively regulates RAS signaling [6].

The large majority (90–95%) of disease-causing *NF1* gene mutations include intragenic mutations, and less than 10% are represented by large deletions encompassing the entire *NF1* gene (whole-gene deletion, WGD) and its flanking genomic regions at 17q11.2 [7,8,9]. So far, four types of WGD are identified, including type-1 deletions of 1.4 Mb, which encompass 14 genes and usually occur as germline mutations, type-2 deletions of 1.2 Mb, which are frequently of post-zygotic origin, type-3 deletions of 1.0 Mb, which include nine genes and are very rare, and atypical *NF1* deletions, which are quite heterogeneous in terms of size and gene content, and may occur as either germline or post-zygotic mutations [9]. Most intragenic mutations are truncating (i.e., frameshift, nonsense, and splice site mutations), or more rarely intragenic number changes (copy number variations, CNVs) (deletions/duplications involving one to multiple exons). A smaller percentage of intragenic mutations are constituted by in-frame variants (i.e., missense changes and in-frame indels involving one to several codons [7,8].

Only a few clinically relevant genotype–phenotype correlations in NF1 are characterized thus far, since they are very difficult to identify because the mutation spectrum is broad, with the majority of individuals having rare/private mutations, the expression of the phenotype is extremely variable, and the disorder is progressive in nature. The first association involves patients heterozygous for germline type-1 large deletions. These patients have worse manifestations. Indeed, they frequently exhibit dysmorphic features, overgrowth/tall-for-age stature, significant delay in cognitive development, congenital heart disease (CHD), and a greater number of cutaneous, subcutaneous, and plexiform NFs as compared to the general NF1 population [9,10]. Moreover, NF1 patients with WGD have a higher risk of developing malignant peripheral nerve sheath tumors [9]. The second genotype–phenotype correlation relates to the presence of a 3-bp deletion (c.2970–2972delAAT) within exon 17 of the *NF1* gene. These patients usually have a milder phenotype characterized by CaLS, SF, and complete absence of any cutaneous, subcutaneous, or superficial plexiform NF [11,12]. The third correlation involves individuals carrying a missense mutation affecting codon 1809 within exon 29 of the gene. These individuals present with a distinct phenotype characterized by multiple CaLS with or without SF and LN, absence of clear cutaneous and/or externally visible plexiform NFs, OPG, and a higher than expected prevalence of short stature, learning disabilities, pulmonary valve stenosis (PVS), and features of Noonan syndrome (NS, MIM #163950) [13,14], a genetic disorder characterized by distinctive facial features, short stature, CHD, bleeding problems, and skeletal malformations [15,16]. A forth genotype–phenotype correlation refers to evidence for a severe phenotype associated with missense mutations affecting codons 844–848, characterized by a higher prevalence of major superficial plexiform and symptomatic spinal NFs, symptomatic or asymptomatic OPG, skeletal abnormalities, and higher predisposition to develop malignancies compared with the general NF1 affected population [17]. Recently, another correlation was identified between the p.Arg1038Gly missense variant and a cutaneous phenotype without neurofibromas or other complications [18]. Clinically related to *NF1* mutations, heterozygous variants in the *SPRED1* gene, encoding another negative regulator of RAS–MAPK signaling, were reported in Legius syndrome, an autosomal dominant disorder that shows some similarities to NF1 but is less severe [19,20].

Previously, we observed an increased prevalence of non-truncating mutations in patients with neurofibromatosis Noonan syndrome (NFNS, MIM #601321), a condition characterized by a clinical phenotype with features overlapping NF1 and NS [21,22]. The majority of NFNS patients are heterozygous for *NF1* gene mutations, generally non-truncating variants—missense changes and in-frame deletions [22]. In a minority of cases, the trait is caused by the concomitant occurrence of mutations in both *NF1* and *PTPN11*, the major gene contributing to NS [15,16], or in another gene mutated in NS, and they are, thus, explained as the coexistence of two diseases [23,24,25,26,27,28]. Molecular genetics increased our knowledge of these conditions, and the collected data support the idea that a subset of mutations in *NF1* is associated with a clinical phenotype that may overlap NS. Indeed, some *NF1* mutations, e.g., c.2970–2972delAAT deletion [11,12,29] or missense changes at codon 1809 [13,14], were reported in both classic NF1 and NFNS. Similarly, an increased proportion of missense/in-frame mutations in individuals with NF1-related PVS [30], most of whom clinically fit with NFNS or Watson syndrome (WTSN #193520), an NF1-related trait with PVS, CaLS, and intelligence at the lower end of the normal change [31,32], was reported.

The prevalence of CHD in individuals with NF1 is not well defined, ranging from 2% to 27%, based on medical histories reported to an international database [33,34] or on an echocardiographic study of clinical series [10,35], respectively. Approximately 50% of NF1 individuals with CHD have PVS [33,34]. Other CHDs often detected in patients with NF1 are aortic stenosis, aortic coarctation, atrial septal defects (ASD), ventricular septal defects (VSD), and hypertrophic cardiomyopathy (HCM) [10,33,34,35]. CHDs and HCM seem to be especially frequent among patients with germline type-1 deletions of the entire *NF1* gene [9,10]. Adults with NF1 may also develop pulmonary hypertension, often in association with parenchymal lung disease, another late-onset and potentially serious complication; intracardiac neurofibromas may also occur [10].

To further evaluate the type and prevalence of CHD in NF1 and better define the clinical and molecular characteristics of NF1 patients with CHD, we retrospectively revised the prevalence and type of cardiac and non-cardiac features, as well the mutation spectrum, in a large series of patients with molecularly confirmed NF1, and correlated the mutation type with the presence and subgroups of CHD.

## 2. Materials and Methods

### 2.1. Study Cohort

The study cohort included a total of 493 individuals with molecularly confirmed diagnosis of NF1, who were referred for genetic testing because of a clinical suspect of the disease, either to the Molecular Diagnostic Laboratory of CSS-Mendel Institute (Rome, Italy) (*n* = 440) or to the Laboratory of Medical Genetics of Bambino Gesù Pediatric Hospital (Rome, Italy) (*n* = 53), and for whom cardiac evaluation data were available. Of these, 67 patients were clinically evaluated at the Medical Genetics Section of Bambino Gesù Pediatric Hospital (Rome, Italy), 120 patients were evaluated at the Department of Translational Medicine, Federico II University, Pediatric Section (Naples, Italy), and the remaining 306 subjects were evaluated at other medical centers in Italy. Clinical data were collected through a shared clinical questionnaire that included family history, sex, age at last follow-up, presence or absence of CaLS, SF, cutaneous and subcutaneous NFs, plexiform NFs, LN, OPG, skeletal malformations, cardiovascular malformations, learning and intellectual disability, cerebrovascular malformations, NS facial features, growth, thoracic anomalies, macrocephaly, urogenital anomalies, hypertension, and occurrence of neoplasms other than NFs or OPG. Informed consent was obtained from all patients or their legal guardians. Clinical and genetic analyses were conducted with the approval of the institutional review boards of the participating institutions (n° 315/18).

### 2.2. Molecular Studies

Mutation analysis was performed by either direct sequencing or parallel sequencing scan of all *NF1* coding exons and their flanking intronic portions (*NF1*, NM_000267.3), combined with multiplex ligation-dependent probe amplification (MLPA) to screen for the presence of *NF1* intragenic deletions/duplications, and WGDs. Parallel sequencing libraries were prepared using either the HaloPlexHS target enrichment kit (Agilent, Santa Clara, CA, USA) or the SureSelect target enrichment system (Agilent, Santa Clara, CA, USA), whereas sequencing was carried out on a MiSeq platform (Illumina, San Diego, CA, USA). Variant calling and data analyses were performed using an in-house implemented pipeline, as previously described [36]. All variants detected by NGS were validated by Sanger sequencing, as previously reported [7]. The MLPA kits no. P081 and P082 (MRC-Holland, Amsterdam, Netherlands) were used to screen for the presence of intragenic deletions or duplications, as previously described [37].

### 2.3. Statistical Analysis

Categorical variables were analyzed and compared using either the chi-square or Fisher’s exact test. Results are expressed as *p*-values. A *p*-value <0.05 was considered significant. The odds ratio, its standard error, and its 95% confidence interval (CI) were calculated according to Reference [38].

## 3. Results

The study cohort included 153 familial cases belonging to 130 apparently unrelated families, 168 sporadic cases, and 172 cases with unknown family history. In total, 217 patients were males and 276 were females. The average age at the time of the observation was 21 years and four months (range: four months to 64 years).

All 493 patients enlisted in the study were heterozygous for an *NF1* variant. Of these, 158/493 (32.0%) had nonsense mutations, 130/493 (26.4%) carried frameshift indels, 74/493 (15.0%) subjects were heterozygous for splice site changes, and 6/493 (1.2%) exhibited intragenic CNVs, whereas 14/493 (2.8%) carried large deletions encompassing the entire gene. Finally, 111/493 (22.6%) subjects had missense variants or in-frame indels. Mutation analysis was performed on germline DNA, and only mutations in the classical splice sites or known to alter splicing were classified as splicing mutations; therefore, the prevalence of this type of mutation could be an underestimation since it is well established that many *NF1* mutations alter splicing through non-canonical splicing sites [39]. Among patients with intragenic mutations, 111/479 (23.2%) had predicted non-truncating mutations and 368/479 (76.8%) had predicted truncating mutations, a finding that is in line with prior studies [8].

### 3.1. Congenital Heart Disease

CHD was present in 62/493 patients (12.6%). Among these, 42/62 (67.8%) were females and 20/62 (32.2%) were males. The prevalence of males and females in patients with CHD was not statistically different from that observed in the total cohort (*p* > 0.05). The most common CHD was PVS, which was diagnosed in 21/62 patients (33.9%). Among individuals with PVS, 15/21 (71.4%) were females and 6/21 (28.6%) were males. Pulmonic valve dysplasia (PVD) without pressure gradient accounted for 2/62 (3.2%) individuals. In one case, PVD was combined with aortic valve dysplasia.

Mitral valve anomalies were reported in 20/62 (32.3%) individuals, mostly represented by cases with mitral valve prolapse (MVP), mitral valve insufficiency (MVI), or both. Aortic valve insufficiency and polyvalvular dysplasia were observed in one case each (1/62, 1.6%). Taken together, valvular heart diseases accounted for 45/62 (72.6%) individuals in the present series. Septal defects were observed in 10/62 (16.1%) patients. Two patients had ASD, two had VSD, and six showed patent foramen ovale (PFO) with minimal left-to-right shunt. One of the patients with VSD had also PFO. Two patients had patent ductus arteriosus (PDA). Dextrocardia, transposition of the great arteries, tetralogy of Fallot, HCM, and lipomatous hypertrophy of the interatrial septum were seen in one patient each. Type and prevalence of CHDs in the study are summarized in Table 1.

**Figure 1:**
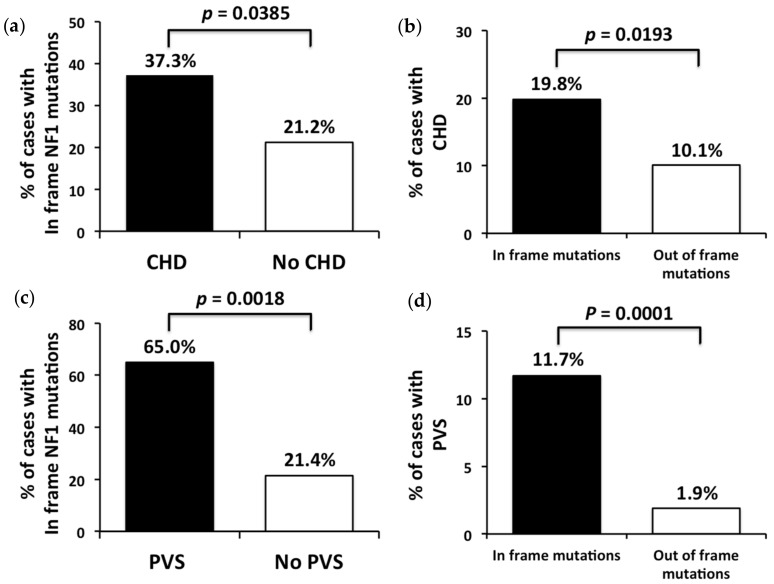
Prevalence of non-truncating mutations in individuals with intragenic *NF1* mutations stratified according to the presence/absence of an unspecified congenital heart disease (CHD), or solely based on the presence/absence of pulmonary valve stenosis (PVS), and prevalence of CHD and PVS in individuals with non-truncating *NF1* intragenic mutations. (**a**) Increased prevalence of *NF1* non-truncating mutations observed among neurofibromatosis type 1 (NF1) individuals with CHD compared with those without CHD. (**b**) Increased prevalence of CHD among NF1 individuals with intragenic non-truncating mutations respect to patients with *NF1* intragenic truncating mutations. (**c**) Increased rate of *NF1* non-truncating mutations among NF1 individuals with PVS respect to those without PVS. (**d**) Prevalence of PVS in individuals with *NF1* intragenic non-truncating mutations respect to those with intragenic truncating mutations.

Among the NF1 patients with CHD, 3/62 (4.8%) carried a WGD and 59/62 (95.2%) had an intragenic mutation. Among patients with intragenic mutations, 22/59 (37.3%) had non-truncating (in-frame) mutations. Remarkably, this type of *NF1* mutation was significantly less represented among the patients with intragenic mutations without CHD (89/420, 21.2%, *p* = 0.038502) (Figure 1a). Consistent with the mutation spectrum of patients with CHD being enriched with in-frame *NF1* intragenic mutations, these patients had more than two-fold higher risk than patients with truncating mutations to develop CHD (CHD prevalence: in-frame mutations: 22/111 (19.8%) vs. truncating mutations: 37/368 (10.1%), odds ratio = 1.9713, 95% CI: 1.1162–3.4814, *p* = 0.0193) (Figure 1b). Individuals with *NF1* WGD also showed higher prevalence of CHD (3/14, 21.4%) compared to patients with intragenic mutations (59/479, 12.3%), but the difference was non-significant (odds ratio = 1.9414, 95% CI: 0.5263–7.1622, *p* = 0.3192), likely because of the small number of patients with these *NF1* lesions. Moreover, patients with CHD showed a significantly higher prevalence of NS-like facial features compared to patients without CHD (*p* < 0.00001), and a relatively higher, but non-significant prevalence of learning disabilities, macrocephaly, thoracic anomalies, and short stature, as well as a non-significant lower prevalence of plexiform neurofibromas (see Table 2 for details).

Of note, among patients with CHD, anomalies of the pulmonary valve were significantly more prevalent in patients with non-truncating mutations (14/22, 63.6%) compared with patients with truncating mutations (8/37, 21.6%) (*p* = 0.033785). Conversely, anomalies of the mitral valve were more frequent in individuals with truncating mutations (17/37, 45.9%) than in those with in-frame mutations (3/22, 13.6%), even though this difference was not significant (*p* = 0.064013) (see Table 3 for details). Interestingly, when excluding patients with PVS from the group of patients with CHD, the prevalence of intragenic in-frame mutation was similar (9/39, 23.1%) to that observed in NF1 patients without CHD (89/420, 21.2%) (*p* > 0.05). However, when we grouped together patients with CHD other than PVS (or without other valve anomalies), e.g., patients with septal defects (ASD (*n* = 2), VSD (*n* = 2), PFO (*n* = 6)), conotruncal (TOF (*n* = 1)), or laterality (TGA (*n* = 1), dextrocardia (*n* = 1)) defects, and with cardiac hypertrophy (*n* = 1), and other cardiac defects (patent ductus arteriosus (PDA) (*n* = 2), lipomatous hypertrophy of the interatrial septum (*n* = 1)), we noticed a slightly higher, but non-significant, prevalence of in-frame changes (5/17, 29.4%) than in patients without CHD (89/420, 21.2%) (*p* > 0.05). Non-cardiac clinical characteristics of NF1 patients with and without CHD are reported in Table 2, whereas the type and frequency of CHDs in patients with intragenic in-frame and out-of-frame mutations, and in NF1 patients with WGD are shown in Table 3.

### 3.2. Pulmonary Valve Stenosis

Pulmonary valve anomalies were diagnosed in 23/493 (4.7%) individuals of the total cohort, and in 23/62 (37.1%) individuals with CHD. In two cases, the pulmonary valve anomaly was combined with an ASD. Among patients with pulmonary valve anomalies, 21 had PVS with or without dysplastic leaflets, whereas two showed pulmonary valve dysplasia without pressure gradient, associated with dysplasia of the aortic valve in one case. Intragenic *NF1* mutations were found in 22/23 (95.7%) patients with pulmonary valve anomalies, whereas one patient harbored a WGD. Similar to NF1 patients with CHD and intragenic mutations, a significantly higher prevalence of non-truncating mutations was observed in patients with PVS (13/20, 65.0%) compared to patients without PVS (98/459, 21.4%) (*p* = 0.00185) (Figure 1c). Of note, prevalence of PVS was more than six-fold higher in patients with non-truncating intragenic mutations (13/111, 11.7%) compared to those with intragenic truncating mutations (7/368, 1.9%), corroborating the strong correlation between intragenic in-frame mutations and PVS (odds ratio = 6.8411, 95% CI: 2.6574–17.6114, *p* = 0.0001) (Figure 1d).

Remarkably, all but one (19/20) patient with PVS had NS facial features. In addition to facial NS characteristics, NF1 patients with PVS showed also a higher, but non-significant prevalence of other NS-related features like learning disabilities, short stature, macrocephaly, and thoracic anomalies. Interestingly, even if lower than in patients with PVS, patients with CHD other than PVS (18/35, 51.4%) also had a high frequency of NS facial features compared with patients without CHD (48/250, 19.2%) (*p* = 0.002175). Non-cardiac clinical characteristics of NF1 patients with and without PVS are reported in Table 4.

## 4. Discussion

The aim of the current study was to describe the prevalence and type of CHDs and the associated mutation spectrum in a large series of NF1 patients, and correlate the mutation type with the presence and subgroups of CHD. Prevalence of CHDs in people with NF1 is not clearly defined, but was estimated to range from 2% to 27% [33,34,35]. In the present study, CHDs were reported in 12.6% of the studied cohort. Consistent with the literature [9,10], patients with WGD were observed to have a higher prevalence of CHD (21.4%) compared with patients with intragenic mutations (12.3%), confirming that heart defects are more common in these patients than in the general NF1 population. Being observed in 33.9% of the patients with CHD and 4.3% of the total cohort, PVS represented the most common cardiac anomaly seen in our series. This preponderance is similar to that reported in other clinical series, providing further evidence that *NF1* mutations predispose to the development of this particular CHD [12,14,22,30,33,34,35,40,41].

**Figure 2:**
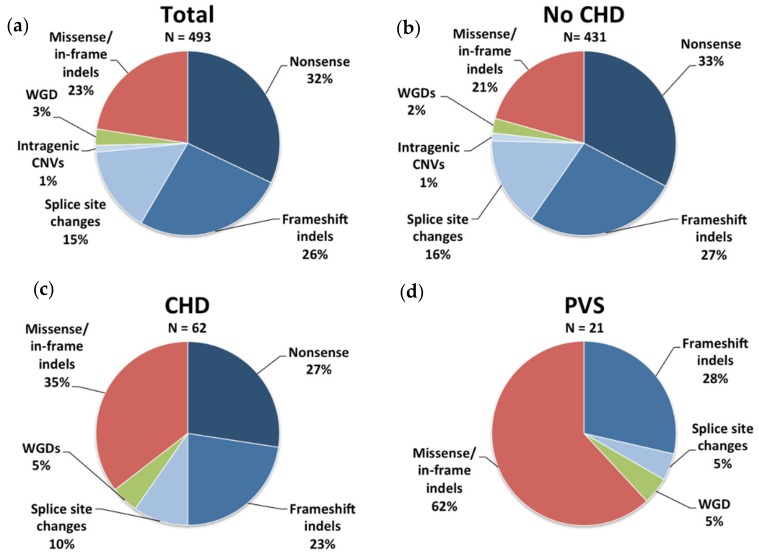
Mutation spectrum of the *NF1* gene in the study cohort. Schematic view of the proportion of nonsense mutations, frameshift indels, splice site changes, intragenic copy number variations (CNVs), whole-gene deletions (WGDs), and missense variants/in-frame indels in (**a**) 493 individuals with diagnosis of NF1, (**b**) 431 individuals with diagnosis of NF1 without CHD, (**c**) 62 individuals with diagnosis of NF1 with CHD, and (**d**) 21 individuals with diagnosis of NF1 with PVS.

The mutation spectrum of NF1 patients with CHD was significantly enriched with non-truncating mutations, missense mutations, and in-frame indels (Figure 2a–d). Of note, when patients were stratified according to the cardiac subgroup, it was evident that non-truncating mutations correlated with PVS, with these patients showing a six-fold higher risk of developing PVS compared with patients with *NF1* out-of-frame mutations, corroborating a previously reported genotype–phenotype correlation [30]. Conversely, the frequency of in-frame lesions in patients with CHD other than PVS overlapped that observed in the general NF1 population.

Being present in 4% of the total cohort and in more than 30% of our patients with CHD, mitral valve anomalies were the second most common cardiac defects in current series. An elevated prevalence of mitral valve anomalies was already reported by other authors [33,35,41]. Remarkably, contrary to subjects with PVS, the majority of subjects with mitral valve anomalies carried truncating *NF1* mutations. Since congenital malformations of the mitral valve affect approximately 0.5% of the general population [42], *NF1* loss of function mutations can be very likely considered a predisposing factor for these cardiac defects. Other valve anomalies were rare in our cohort, and included two cases with PVD without pressure gradient, a case of polyvalvular dysplasia in a patient with WGD, and a single case of aortic valve insufficiency.

The high predominance of PVS and other valve anomalies in NF1 is not unexpected and may have an explanation in the role of neurofibromin in the myocardium. Indeed, studies on mice showed that, in the absence of neurofibromin, mouse embryo hearts develop enlarged and abnormal endocardial cushions due to hyperproliferation and lack of normal apoptosis [43]. Nevertheless, the pathogenic mechanisms via which *NF1* mutations cause PVS should be different from the one causing mitral valve anomalies, since their associated mutational spectra are different, being enriched with in-frame changes in patients with PVS and with truncating mutations in individuals with anomalies of mitral valve (Table 3).

In the National Neurofibromatosis Foundation International Database (NNFID), a repository for collecting comprehensive information on the clinical manifestations and natural history of neurofibromatosis [44], left heart obstruction defects (aortic stenosis or coarctation) were the second most prevalent CHD, representing approximately 15% of the CHDs [33]. However, the authors reported that, in most NF1 patients, aortic arch narrowing was of the fusiform type, which is anatomically different from the segmental constriction that is seen in patients without NF1 [33,34]. In the present series, we did not observe any case with left heart obstruction defects, suggesting that further and accurate studies on larger series are necessary to determine the prevalence and subtypes of these cardiac defects in NF1.

One patient of the present series was reported to have isolated HCM, while two other individuals showed a mild septal hypertrophy in conjunction with either mitral valve insufficiency or polyvalvular dysplasia. Out of these three cases, one had a WGD and two showed a truncating mutation. Although HCM was not observed in patients from the NFFID database [33], this cardiac anomaly seems to be more frequent in NF1 patients with WGD. In addition to what observed in current series, a precedent study systematically assessing 16 patients with *NF1* WGD by echocardiography identified three patients who developed HCM [10]. Consistent with a possible role of *NF1* mutations in HCM, conditional knock-out mice lacking the murine ortholog, *Nf1*, in myocardium, were shown to have normal embryonic cardiovascular development but marked cardiac hypertrophy, progressive cardiomyopathy, and fibrosis in the adult, together with hyperactivation of Ras and downstream pathways [45]. It is plausible that HCM is rare in the general NF1 population, but more common in NF1 patients with WGD.

Similarly to that observed in the NNFID [33,34] and other series [35,41], complex CHDs (e.g., conotruncal, atrioventricular, laterality, and looping defects) were uncommon in the present cohort, overall representing about 5% of the CHDs of our patients (4% in the NNFID database). Conversely, septal defects with left-to-right shunts (VSD, ASD, PFO, and PDA) were more frequent, being responsible for approximately 20% of the CHD cases in present study and NNFID cohort [33]. It is of note that the two subjects with VSD and one of the two cases with ASD harbored in-frame alleles.

Although the most striking finding of this study was the association between PVS and *NF1* in-frame changes, the existence of other genotype–phenotype correlations cannot be excluded. Grouping together all patients with cardiac structural anomalies other than those affecting valves (conotruncal, laterality, septal defects, and HCM), we noticed that the frequency of *NF1* in-frame changes was slightly higher compared with that of patients without CHD. The present cohort has limited statistical power to detect small associations; therefore, we cannot exclude that the same *NF1* mutations that predispose to PVS could also predispose, to a lesser extent, to other CHDs (e.g., left-to-right shunts such as VSD or ASD).

PVS is the characteristic CHD of patients with NFNS [21]. In the present cohort, almost all patients with PVS and, to a lesser extent, those with other CHDs showed NS-related features, such as short stature, macrocephaly, and thoracic anomalies (see Table 2 and Table 4 for details). An association between NFNS, high risk of developing PVS, and high prevalence of non-truncating *NF1* mutations was previously described by our group [22] and others [29,30,46,47,48,49]. We previously reported that a significant number of these in-frame changes are located within the neurofibromin GAP-related domain [22]. Considering that PVS is a classic cardiac feature of NS, the elevated prevalence of PVS in patients with *NF1* in-frame mutations, together with the increased incidence of NS-like related features in NF1 patients with PVS, favors the hypothesis that some *NF1* specific mutations may predispose more toward an NS-like phenotype rather than to a classic NF1 phenotype. Indeed, patients with NFNS generally tend to show less penetrance for neurofibromas, the typical hallmark of NF1 [21,22]. This concept seems to be consistent with the recent identification of specific genotype–phenotype correlations in NF1, including the high incidence of NS facial features, short stature, PVS, and learning disabilities, and the absence of clear cutaneous and/or externally visible plexiform NFs and OPG in patients with *NF1* missense mutations affecting p.Arg1809 [13,14], together with the identification of families segregating the NFNS phenotype [11,22,29], as well as the identification of the p.Met1035Arg change in a patient with multiple lentigines syndrome (LEOPARD syndrome, LPRD1, MIM #151100) [50], another NS-related disorder. NF1 belongs to RASopathies, a clinically defined group of genetic syndromes caused by germline mutations in genes that encode components or regulators of the Ras/mitogen-activated protein kinase (RAS–MAPK) pathway. It was previously proposed that, like the other RASopathies, the increased predisposition of NF1 patients to develop PVS is likely related to the role of neurofibromin in the RAS–MAPK signaling pathway [30]. In particular, it was hypothesized that truncating and non-truncating *NF1* mutations may contribute in a similar manner to some aspects of the disease, e.g., neurofibroma development, but may have different roles in other aspects of the disease, e.g., predisposition to PVS or other NS-related features [30].

## 5. Conclusions

Present data confirm the significant frequency of CHD in patients with NF1, mostly represented by pulmonary valve stenosis and mitral valve anomalies, and more rarely by septal defects or other CHDs. Moreover, they clearly show that the majority of NF1 patients with PVS display NS-like characteristics and have a higher than expected frequency of in-frame *NF1* variants, whereas NF1 patients with mitral valve anomalies have a mutation spectrum overlapping that of the NF1 general population. This evidence confirms that all children with NF1 should have a careful cardiac examination, in particular those showing signs of NFNS and/or harboring mutations that are consistently associated with either NFNS and/or PVS. In parallel, current findings confirm the importance of persisting in searching for genotype–phenotype correlations in NF1, favoring the use of large and clinically well-characterized cohorts, as well as studies aimed at dissecting the effects of specific mutations on neurofibromin functioning.

## Figures and Tables

**Table 1:** Type and frequency of congenital heart diseases (CHDs) in present cohort.

CHDs (*n* = 62/493, 12.6%)	CHD Feature, *n* (%)
Pulmonary valve stenosis ^a^ (PVS)/dysplasia (PVD) (total)	23/62 (37.1)
• PVS ^b^	19/23 (82.6)
• PVS plus atrial septal defects (ASD)	2/23 (8.7)
• PVD	1/23 (4.3)
• PVD plus aortic valve dysplasia	1/23 (4.3)
Mitral valve anomalies (total)	20/62 (32.3)
• Mitral valve prolapse (MVP)	5/20 (25)
• Mitral valve insufficiency (MVI)	4/20 (20.0)
• MVP and tricuspid vale insufficiency (TVI)	3/20 (15.0)
• MVP and MVI	2/20 (10.0)
• Mitral valve dysplasia (MVD)	1/20 (5.0)
• Mild MVD plus bicuspid aortic valve	1/20 (5.0)
• MVI and arching of the posterior mitral leaflet	1/20 (5.0)
• MVI and mild septal hypertrophic cardiomyopathy	1/20 (5.0)
• Mitral valve thickening	1/20 (5.0)
• Mild arching mitral valve	1/20 (5.0)
Polyvalvular dysplasia ^c^	1/62 (1.6)
Mild aortic valve insufficiency with normal tricuspid aortic valve	1/62 (1.6)
Patent ductus arteriosus (PDA) ^d^	2/62 (3.2)
ASD ^e^	2/62 (3.2)
Ventricular septal defects (VSD) ^f^	2/62 (3.2)
Patent foramen ovale (PFO)	6/62 (9.7)
Transposition of the great arteries	1/62 (1.6)
Dextrocardia ^h^	1/62 (1.6)
Tetralogy of Fallot	1/62 (1.6)
Hypertrophy of the left ventricle	1/62 (1.6)
Lipomatous hypertrophy of the interatrial septum	1/62 (1.6)
CHDs (*n* = 62/493, 12.6%)	CHD feature, *n* (%)

^a^ ±dysplasia; ^b^ with PVD, mild MVP, and MVI in one case; ^c^ with mild interventricular septal hypertrophy; ^d^ with PFO in one case; ^e^ ostium secundum type in one patient; ^f^ with PFO in one patient; ^h^ with MVP.

**Table 2:** Prevalence of non-cardiac phenotypic characteristics in neurofibromatosis type 1 (NF1) patients of present series, with and without CHD.

	CHD (*n* = 62)	No CHD (*n* = 431)	Chi-Square Significance
Sex	20 M, 42 F	198 M, 233 F	
Age at observation	12 y 7 m, max = 54 y, min = 4 m	22 y 10 m, max = 64 y, min = 4 m	
CaLS	61/61 (100.0%)	276/277 (99.6%)	*p* > 0.05
Freckling	51/60 (83.6%)	205/269 (76.2%)	*p* > 0.05
Cutaneous NF ^a^	5/9 (55.6%)	107/143 (74.8%)	*p* > 0.05
Subcutaneous NF ^a^	4/7 (57.1%)	78/133 (58.6%)	*p* > 0.05
Plexiform NF ^b^	4/29 (13.8%)	51/168 (30.4%)	*p* > 0.05
Lish nodules	23/50 (46.0%)	89/206 (43.2%)	*p* > 0.05
Optic glioma	8/41 (19.5%)	29/180 (16.1%)	*p* > 0.05
Skeletal dysplasia	0/52 (0.0%)	19/252 (7.5%)	*p* > 0.05 ^c^
Scoliosis	20/60 (33.3%)	70/270 (25.9%)	*p* > 0.05
Learning disabilities	23/56 (41.1%)	62/225 (27.6%)	*p* > 0.05
Intellectual disability	6/59 (10.2%)	23/229 (10.0%)	*p* > 0.05
Macrocephaly	9/19 (47.4%)	26/110 (23.6%)	*p* > 0.05
**NS facial features**	**37/55 (64.9%)**	**48/250 (19.2%)**	***p* < 0.00001**
Thoracic anomalies	4/27 (14.8%)	13/250 (5.2%)	*p* > 0.05
Urogenital anomalies	0/24 (0.0%)	3/209 (1.4%) ^c^	*p* > 0.05 ^d^
Short stature	22/57 (38.6%)	42/150 (28.0%)	*p* > 0.05
Hypertension	2/54 (3.7%)	14/256 (5.5%)	*p* > 0.05
Vasculopathy	2/40 (5.0%)	7/46 (15.2%)	*p* > 0.05
Other neoplasms	7/48 (14.6%)	31/194 (16.0%)	*p* > 0.05

^a^ In individuals ≥19 years old; ^b^ in individuals ≥9 years old; ^c^ all patients with urogenital anomalies had NS facial features; ^d^ Fisher exact test. M, male; F, female; m, months; y, years; max, maximum; min, minimum; CaLS, café-au-lait spots; NF, neurofibromas; NS, Noonan syndrome. Statistically significant associations are evidenced in bold.

**Table 3:** Type and frequency of CHDs in patients with intragenic in-frame and out of frame mutations, and in NF1 patients with whole-gene deletion (WGD).

CHDs (*n* = 62/493, 12.6%)	Intragenic *NF1* Mutations		WGD, *n* = 3 (%)
	In-Frame Mutations, *n* = 22 (%)	Out-of-Frame Mutations, *n* = 37 (%)	
Pulmonary valve stenosis ^a^ (PVS)/dysplasia (PVD) (total)	14/22 (63.6) *	8/37 (21.6) *	1/3 (33.3)
• PVS	13/14 (92.9)	5/8 (62.5) ^b^	1/1 (100)
• PVS plus ASD		2/8 (25.0)	
• PVD		1/8 (12.5)	
• PVD plus aortic valve dysplasia	1/14 (7.1)		
Mitral valve anomalies (total)	3/22 (13.6) **	17/37 (45.9) **	0/3 (0.0%)
• Mitral valve prolapse (MVP)	1/3 (33.3)	4/17 (23.5)	
• Mitral valve insufficiency (MVI)	1/3 (33.3)	4/17 (23.5)	
• MVP and tricuspid valve insufficiency (TVI)		1/17 (5.9)	
• MVP and MVI	1/3 (33.3)	2/17 (11.8)	
• Mitral valve dysplasia (MVD)		1/17 (5.9)	
• Mild MVD plus bicuspid aortic valve		1/17 (5.9)	
• MVI and arching of the posterior mitral leaflet		1/17 (5.9)	
• MVI and mild septal hypertrophic cardiomyopathy		1/17 (5.9)	
• Mitral valve thickening		1/17 (5.9)	
• Mild arching mitral valve		1/17 (5.9)	
Polyvalvular dysplasia			1/3 (33.3) ^c^
Mild aortic valve insufficiency with normal tricuspid aortic valve		1/37 (2.7)	
Patent ductus arteriosus		2/37 (5.4) ^d^	
Atrial septal defects (ASD)	1/22 (4.5)	1/37 (2.7) ^e^	
Ventricular septal defects	2/22 (9.1) ^f^		
Patent foramen ovale (PFO)	1/22 (4.5)	4/37 (10.8)	1/3 (33.3)
Transposition of the great arteries		1/37 (2.7)	
Dextrocardia		1/37 (2.7) ^h^	
Tetralogy of Fallot		1/37 (2.7)	
Hypertrophy of the left ventricle		1/37 (2.7)	
Lipomatous hypertrophy of the interatrial septum	1/22 (4.5)		

* Pulmonary valve anomalies were significantly more prevalent in patients with intragenic non-truncating mutations with respect to patients with truncating mutations (*p =* 0.033785); ** mitral valve anomalies were more frequent in individuals with truncating mutations respect to those with in-frame mutations, although the difference was not significant (*p =* 0.064013); ^a^ ±dysplasia; ^b^ with PVD, mild MVP, and mild MVI in one case; ^c^ with mild interventricular septal hypertrophy; ^d^ with PFO in one case; ^e^ ostium secundum type; ^f^ with PFO in one patient; ^h^ with MVP.

**Table 4:** Prevalence of non-cardiac phenotypic characteristics in NF1 patients of present series, with and without PVS.

	PVS (*n* = 21)	No PVS (*n* = 472)	Chi-Square Values
Sex	6 M, 15 F	212 M, 260 F	
Age at diagnosis	9 y 6 m, max = 33 year, min = 2 year	21 y 11 m, max = 64 year, min = 4 min	
CaLS	21/21 (100.0%)	316/317 (99.7%)	*p* > 0.05
Freckling	17/21 (81.0%)	239/308 (77.6%)	*p* > 0.05
Cutaneous NF ^a^	1/1 (100.0%)	110/150 (73.3%)	*p* > 0.05 ^c^
Subcutaneous NF ^a^	1/1 (100.0%)	81/139 (58.3%)	*p* > 0.05 ^c^
Plexiform NF ^b^	0/9 (0.0%)	53/186 (28.5%)	*p* > 0.05 ^c^
Lish nodules	4/17 (23.5%)	108/240 (45.0%)	*p* > 0.05
Optic Glioma	3/13 (23.1%)	34/208 (16.3%)	*p* > 0.05
Skeletal dysplasia	0/16 (0.0%)	19/289 (6.6%)	*p* > 0.05 ^c^
Scoliosis	6/20 (30.0%)	84/310 (27.1%)	*p* > 0.05
Learning disability	11/20 (55.0%)	74/261 (28.4%)	*p* > 0.05
Intellectual disability	1/21 (4.8%)	28/267 (10.5%)	*p* > 0.05 ^c^
Macrocephaly	4/8 (50.0%)	31/121 (25.6%)	*p* > 0.05
**NS facial features**	**19/20 (95.0%)**	**66/285 (23.2%)**	***p* = 0.000018**
Thoracic anomalies	2/13 (15.4%)	14/264 (5.3%)	*p* > 0.05
Urogenital anomalies	0/12 (0.0%)	3/221 (1.4%)	*p* > 0.05 ^c^
Short stature	10/18 (55.6%)	54/189 (28.6%)	*p* > 0.05
Hypertension	0/18 (0.0%)	17/292 (5.8%)	*p* > 0.05 ^c^
Vasculopathy	0/11 (0.0%)	9/75 (12.0%)	*p* > 0.05 ^c^
Other neoplasms	3/19 (15.8%)	36/223 (16.1%)	*p* > 0.05

^a^ In individuals ≥19 years old; ^b^ in individuals ≥9 years old; ^c^ Fisher exact test; M, male; F, female; m, months; y, years; max, maximum; min, minimum. Statistically significant associations are evidenced in bold.

## Supplementary Materials

Supplementary File 1
